# Use of Artificial Intelligence in the Interpretation of Electroretinography (ERG) Studies

**DOI:** 10.3390/ijms27083491

**Published:** 2026-04-14

**Authors:** Manasi Hegde, Alexander Thomis, Sheetal Shirke

**Affiliations:** 1 Department of Ophthalmology, Townsville University Hospital, 100 Angus Smith Drive, Douglas, Townsville, QLD 4814, Australia; manasi.hegde@health.qld.gov.au (M.H.); alexander.thomis@health.qld.gov.au (A.T.); 2 Faculty of Medicine, University of Queensland, Herston, Brisbane, QLD 4006, Australia

**Keywords:** artificial intelligence, electroretinograms, diagnostic accuracy

## Abstract

Electroretinograms are an important diagnostic tool to measure retinal electrical activity. However, their interpretation, done by sub-specialised ophthalmologists, can be not only time consuming but also challenging to obtain due to availability. In recent years, studies have investigated the use of artificial intelligence in the analysis of electroretinograms. This systematic review summarises the accuracy of artificial intelligence in interpreting electroretinograms and appraises the studies included. The review comprises primary, peer-reviewed published studies that determined accuracy of artificial intelligence by comparison to an expert ophthalmologist. In the 14 studies retrieved from databases and published between 2006 and 2025, machine learning was the most widely used artificial intelligence, with an accuracy rate between 39.3% and 100%. Overall, the “artificial neural network” machine learning tool was the most accurate. Quality assessment of the studies demonstrated high bias in patient selection but robustness in the methodology for the reference standard, flow and timing. The results revealed potential benefits in the real-world use of artificial intelligence in ophthalmic diagnostic testing; however, the variability in results suggests a requirement for further investigation prior to clinical implementation.

## 1. Introduction

The electroretinogram (ERG) measures electrical activity of the retina in response to light stimulation [1,2]. Summated cellular signals from different layers of the retina are recorded as a trace. Specifically, phototransduction leads to hyperpolarisation of the retina’s outermost layer, the photoreceptor layer, which contains rod and cone cells [3]. Photoreceptor hyperpolarisation is represented as a negative “a-wave” form in the ERG [4]. In turn, there is a decrease in glutamate release from the photoreceptors to the post-synaptic bipolar cells, located in the inner nuclear layer of the retina [4,5]. Resulting depolarisation of bipolar cells signifies a positive “b-wave” in the ERG [4,5]. Finally, action potentials are produced in post-synaptic cells of the retinal ganglion cell layer. Oscillatory potentials are an ERG wave form representative of the ganglion cell activity [4]. Understanding the cellular components of this electrical circuit can assist in the diagnosis of dysfunction along this pathway.

The clinical utility of ERGs in early diagnosis of ophthalmic conditions is well documented, especially its ability to detect disease where there is no structural change shown on other imaging modalities [6]. It is essential for the diagnosis of a range of retinal disorders, and may also be used to monitor disease progress in dystrophies, forms of uveitis and drug toxicity [1,7,8]. Its role in targeting ganglion cell function, such as in optic neuropathy and early glaucoma, has also been established [1]. Three major types of ERGs exist: full-field (ffERG), multifocal (mfERG) and pattern (PERG), for which clinical applications differ [9].

ERG interpretation is performed by trained ophthalmologists in clinical practice [10]. The process can be time-consuming, and ophthalmologists with the necessary training are not always widely available [10,11]. Accurate diagnosis and staging are key to treatment and controlling disease progression, but can be delayed significantly by resourcing constraints [10].

Artificial intelligence (AI) methods such as machine learning and deep learning have increasingly been explored to aid clinical diagnosis in several fields of medicine, in many instances achieving expert-level performance [10,11,12]. In ophthalmology, which is heavily reliant on specialised investigations in various modalities, the scope for augmentation with computer-aided diagnosis is substantial [13]. Research has demonstrated the capability of machine and deep learning technologies in detecting and staging diseases such as diabetic retinopathy and glaucoma [14,15,16]. Optical coherence tomography, fundus photography and visual field testing are examples of investigations for which interpretation using AI has shown promise [13,15].

Relatively few studies, however, have assessed the applicability of AI to visual electrophysiology. To date, no systematic review has evaluated evidence on the potential role of AI technologies as a diagnostic aid in electroretinography.

This review aims to identify, appraise and synthesise primary literature assessing the accuracy of AI methods in the interpretation of ERG studies.

## 2. Materials and Methods

### 2.1. Protocol

**Figure 1:**
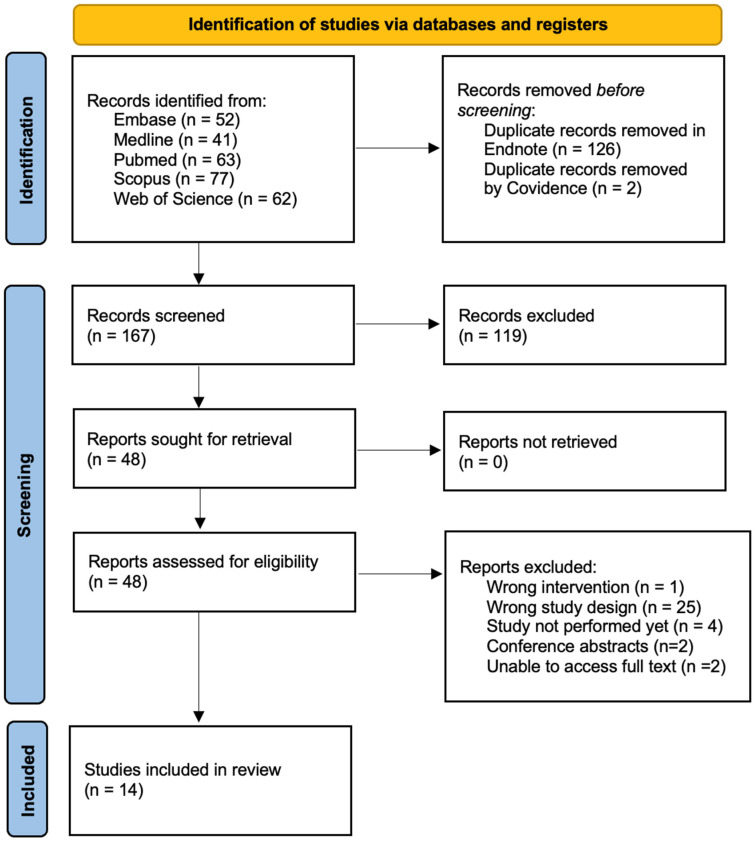
PRISMA 2020 flow diagram of the study [17].

This systematic review was conducted in accordance with the PRISMA (Preferred Reporting Items for Systematic Reviews and Meta-Analyses) framework (see Figure 1) [17]. The PRISMA checklist can be found in the Supplementary Materials. This study was prospectively registered in PROSPERO, and the protocol can be accessed through study ID: CRD420261327277.

### 2.2. Literature Search Strategy

A comprehensive literature search was conducted across PubMed, Medline, Embase, Scopus and Web of Science international databases, from inception to present. Keywords in the search strategy included, but were not limited to, “artificial intelligence”, “machine learning”, “deep learning”, “computational neural network”, “predictive learning models”, “electroretinography” and “electroretinogram”. Keywords were combined using the Boolean operators “OR” and “AND”, as detailed in Supplementary Digital Content Appendix A. The systematic search was performed independently by two researchers (M.H. and A.T.).

### 2.3. Eligibility Criteria

The following inclusion criteria were defined:

- Primary research, including comparative, retrospective and clinical research.
- Publication in a peer-reviewed journal.
- Use of artificial intelligence (AI) in the evaluation of electroretinography studies.
- Comparison to ERG evaluation by expert reviewers (Ophthalmologists).
- Studies conducted across all dates, populations and languages.

The following exclusion criteria were defined:

- Abstracts without full-text availability.
- Use of electrophysiology testing other than electroretinography, such as visual evoked potentials.
- Reviews, conference papers, editorials, author-responses, theses and books.

### 2.4. Data Extraction and Risk of Bias Assessment

Covidence was used for data extraction and reporting bias. This was completed by two reviewers (M.H. and A.T.) independently, with discussion to resolve conflicts. The following data were extracted from each study: first author, year of publication, study location (country), study design, population (human or non-human, adult, paediatric, gender), mean age (years), disease for diagnosis, type of AI and key findings (accuracy or mean error of the tool). Findings were standardised across studies. Finally, risk of bias was assessed using the QUADAS-2 tool [18]. Developed for diagnostic accuracy studies, this tool assesses the risk of bias in patient selection, the index test, reference standard and flow and timing [18].

## 3. Results

### 3.1. Study Selection

The initial search generated 295 studies across the electronic databases. The search consisted of studies up until 24 November 2025 (date of search).

Data management for this study was conducted using EndNote version 21 and Covidence. Initially, duplicates were removed using the EndNote “Find Duplicates” function, with manual confirmation. Then, once imported into Covidence, two duplicates were removed.

Two independent researchers (M.H. and A.T.) conducted the search according to the inclusion and exclusion criteria. Initial screening of studies was based on title and abstract. Eligible papers were then assessed by full-text reviews. Any conflicts were resolved by discussion and consensus between investigators. One study initially appeared to meet inclusion criteria, but on closer analysis was found to evaluate visual evoked potentials predominantly, rather than ERGs [19].

Following implementation of the inclusion and exclusion criteria, this systematic review contained 14 studies. This selection process is illustrated in Figure 1.

### 3.2. Study Characteristics

A total of 14 studies were included in this review, and this is summarised in Table 1. Of note, two studies were conference presentations only and the full text was inaccessible for two studies [20,21,22,23]. Publication dates were between 2006 and 2025. Noting two studies had inadequate information, the total population in this review was 2997 people. Most studies were conducted in Turkey (5/14), followed by Russia (4/14). Where adequate data was available, weighted mean and standard deviations were performed to encapsulate the population of the study. Only eight of the studies reported age of the participants and they all contained adults with an average age range between 35 and 47 years. While most of the papers were retrospective cohort studies (8/14), there were three prospective cohort studies and one randomised controlled trial. The most commonly used ERG modality was the full-field ERG (7/14). Only two studies used deep learning AI tools, compared to 12 with machine learning AI. Finally, a wide range of conditions were used to test for diagnostic accuracy of the AI tool; however, the majority (5/14) assessed for healthy and unhealthy ERGs.

### 3.3. Artificial Intelligence Accuracy

The results of the 14 included studies, presented in Table 1, had varied accuracy in the interpretation of ERGs. Only one study found a diagnostic accuracy of 100% by machine learning technology. Specifically, this study used artificial neural networks to diagnose achromatopsia and congenital stationary night blindness [24]. Similarly, artificial neural networks performed well in other studies demonstrating accuracy rates of 92% and 94.2% when diagnosing optic neuritis [28,29]. Support vector machine learning comprised the next most utilised technology. Within one study, there were wide-ranging accuracy rates, where interpretation of macular dysfunction alone and cone and rod dysfunction was highly accurate at 96.7% and 93.8% respectively, while combined macular and cone dysfunction had detection rates as low as 39.3% [11]. An additional study using support vector machine learning demonstrated reasonable precision at 85.3% [27]. Furthermore, the same machine learning approach outperformed other tools within one study, achieving a positive prediction rate of 87.1% [30]. Subsequently, these authors conducted a further study with an equivalent cohort of retinitis pigmentosa participants, and found naïve Bayes machine learning to outperform the support vector machine with a diagnostic accuracy of 82.32% [31]. In a similar cohort of retinitis pigmentosa patients but by different authors, resnet50 was superior to other machine learning tools within the study at 94.9% accuracy [10]. Alternatively, time series forest machine learning had the highest accuracy rate (74%) when diagnosing optic neuropathy in a study evaluating several machine learning tools [25]. When assessing broadly healthy and unhealthy adults and children, machine learning combined with multi-layered signal processing (wavelet 1–4) achieved the highest accuracy in the adult group (83%); in contrast to the paediatric group that achieved, at best, 70% accuracy from machine learning combined with low-level signal processing (wavelet 1–2) [34]. The same author team used decision tree machine learning and had the lowest diagnostic rate, 52% in adults and 40% in children, across all studies in this systematic review [35]. A study found two machine learning groups to have impressive accuracy rates of 95% when diagnosing normal physiology on high noise artefact ERGs [26]. This was superior to the human electrophysiologist who, in comparison to minimal artefact ERGs, had an accuracy rate of 62% [26]. There were only two studies by the same author group that used deep learning tools. They found the greatest diagnostic precision by vision transformer deep learning tool at 84% and 88%, 84.9% and 85%, and 87.5% and 91% for interpreting maximum ERG responses, scotopic ERG responses, and photopic ERG responses respectively [32,33].

### 3.4. Quality Assessment

**Figure 2:**
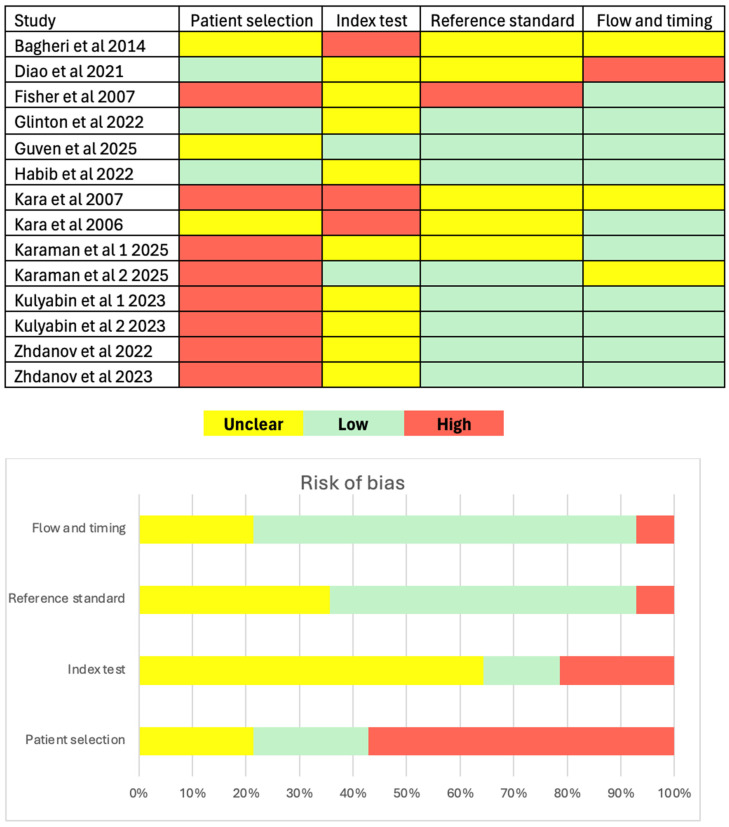
Risk of bias assessment of the studies [10,11,24,25,26,27,28,29,30,31,32,33,34,35] using QUADAS-2 tool [18]. Bias displayed as Unclear (yellow), Low (green) and High (red).

The risk of bias in the methodology for each study is displayed in Figure 2. In terms of patient selection, 57% (8/14) of studies had a high risk of bias and the most common explanations for this classification were lack of random or consecutive sampling and absence of inclusion and exclusion criteria. Only 21% (3/14) of studies achieved low bias for patient selection. Most studies (64%) had unclear index testing bias due to deficient information on blind evaluation. Conversely, high index test bias was seen in 21% (3/14) and low bias was seen in 14% (2/14). Bias of reference standard was much reduced, with 57% (8/14) exhibiting low bias and only 7% (1/14) with high bias. About 71% (10/14) of studies had low bias in terms of flow and timing. Consequently, only 7% (1/14) had high bias in this classification. No single study achieved low bias across all criteria. However, three studies had low bias in three categories and an unclear status in the fourth.

## 4. Discussion

### 4.1. Results Interpretation

To the best of the authors’ knowledge, this is the first systematic review on the evaluation of AI in ERG interpretation. Additionally, this is corroborated by the PROSPERO database. In this systematic review, the machine learning tool, “artificial neural network” had the most impressive rates of accuracy compared to other machine learning and deep learning apparatus. Similar high-accuracy outputs by “artificial neural network” have been demonstrated in other fields of diagnostic medicine [36,37]. However, our findings indicate that there was a wide range of diagnostic accuracy in terms of AI interpretation of ERGs. This differs from some of the more established uses of AI in ophthalmic diagnostics, where results are more consistent. AI detection of diabetic retinopathy on fundus imaging has achieved an average accuracy of 93.6% across six countries [38]. To diagnose keratoconus, AI models have obtained precision between 91.9% and 98.9% [39]. Additionally, glaucoma detection had consistent results and accuracies of between 76% and 98.3% with machine learning tools such as “support vector machine”, “naïve Bayes” and “decision trees” [40].

Moreover, when deep learning AI has been used to interpret other electrophysiology tests, such as cardiac electrophysiology, it has achieved cardiologist-level performance with arrhythmia detection accuracy in over 95% [41]. Unlike electrocardiograms, however, ERGs are highly susceptible to noise interference making interpretation more challenging [6]. Due to the small amplitude received from the retinal and biologic artefact created by eyelid and facial muscles, noise is a significant limitation in the assessment of ERGs [42]. This could explain the current discrepancies in AI accuracy and encourage ERG de-noising techniques to improve reliability [6]. Alternatively, as seen in one of the included studies, AI can be trained using highly noise-contaminated ERGs to achieve lower error rates than human experts [26].

Electroencephalograms are analogous to ERGs in terms of their need for specialised interpretation and high susceptibility for noise artefact [43]. Despite this, an AI model has been able to correctly classify a large cohort of electroencephalograms with an accuracy of 88.3% [43]. Previous AI models in this field had insufficient precision for clinical implementation [44]. Translated to ERGs, this suggests that continued development of AI interpretation models and assessment of larger cohorts is necessary.

Implementation of AI in medical diagnostics has demonstrated increased efficiency, alleviated workforce burden and optimised patient health outcomes [45]. With development of AI models in future research, ERGs and electrophysiologists will likely reap the same benefits.

### 4.2. Strengths and Limitations

This systematic review was comprehensive, with a predefined search strategy across five international databases. While having a clearly outlined inclusion and exclusion criteria, it remained thorough by including all dates, all languages and human and non-human subjects. The search, study selection, data extraction and quality assessment were conducted independently by two reviewers, increasing the credibility of the findings.

There was significant heterogeneity across the included studies which limited this review. The populations varied between adults and children; additionally, two studies lacked information regarding the population. While broadly, machine learning was most frequently implemented, the subset AIs used were of wide variability and this is also reflected in the accuracy outcomes. As seen in other systematic reviews on the use of AI in ophthalmology, most studies contained patient sampling bias and were frequently unclear regarding blinding [46,47]. With substantial overlap in authors across studies, this increased the risk of publication bias and impaired the generalisability of results. Additionally, only one randomised controlled trial met criteria for the review, highlighting the limited quality of the studies included. The integrity of the review was influenced by small sample sizes, where most studies had less than 150 participants. These limitations highlight the need for further studies on this subject.

## 5. Conclusions

This review demonstrated promise in the use of AI as a diagnostic tool for interpreting ERGs. In comparison to other fields where AI has been successfully implemented, the studies in this review contain high variability. In ophthalmology, ERGs are an objective test that can make a diagnosis at the cellular level. Given the capacity of AI to achieve precision, reduce workforce burden and impact patient care, our recommendation is for further studies assessing the accuracy of AI in the interpretation of ERGs.

## Figures and Tables

**Table 1:** Summary of study characteristics.

First Author	Year	Country	Study Type	Population	Mean Age (Years)	Disease for Diagnosis	Type of ERG Modality	Type of Artificial Intelligence	Key Findings
Bagheri et al. [24]	2014	Italy; United States of America	Retrospective cohort study	Adult participants *n* = 94 Males = 42/94 Females = 52/94	47 +/− 5	Achromatopsia; Congenital stationary night blindness	Full-field ERG	Machine learning	Diagnostic accuracy of the *artificial neural network* machine learning tool was 100%
Diao et al. [25]	2021	United States of America	Case-control study	Adult participants *n* = 119 Males = 56/119 Females = 63/119	45.6 +/− 17.5	Optic neuropathy	Full-field ERG	Machine learning	Highest diagnostic accuracy was by *time series forest* machine learning at 74%
Fisher et al. [26]	2007	United Kingdom	Cluster randomised controlled trial	Human participants *n* = 10	Unstated	Normal physiology	Pattern ERG	Machine learning	Diagnostic accuracy was achieved at a rate of 95% in two machine learning groups compared to 62% in the human group interpreting noisy ERGs
Glinton et al. [11]	2022	United Kingdom	Retrospective cohort study	Human participants *n* = 597 Group 1 = 344/597 Group 2 = 44/597 Group 3 = 209/597	Group 1 = 35 Group 2 = 35 Group 3 = 37	Group 1 = Macular dysfunction alone Group 2 = Macular dysfunction + generalised cone dysfunction Group 3 = cone and rod dysfunction	Full-field ERG	Machine learning	Diagnostic accuracy of *support vector machine* Group 1 = 96.7% Group 2 = 39.3% Group 3 = 93.8%
Guven et al. [10]	2025	Turkey	Case-control study	Adult participants *n* = 206 Males = 118/206 Females = 88/206	35.37 +/− 15.01	Retinitis pigmentosa	Multifocal ERG	Machine learning	Highest diagnostic accuracy was by *resnet50* machine learning at 94.9%
Habib et al. [27]	2022	Canada; China	Retrospective cohort study	Human participants *n* = 748	Unstated	Hydroxychloroquine retinopathy	Multifocal ERG	Machine learning	Diagnostic accuracy of the *support vector machine* machine learning was 85.3%
Kara et al. [28]	2007	Turkey	Prospective cohort study	Adult participants *n* = 320 Males = 164/320 Females = 156/320	41.94	Optic neuritis	Pattern ERG	Machine learning	Diagnostic accuracy of the *artificial neural network* machine learning tool was 92%
Kara et al. [29]	2006	Turkey	Prospective cohort study	Adult participants *n* = 256 Males = 117/256 Females = 119/256 Unspecified gender = 20/256	43.8	Optic neuritis	Pattern ERG	Machine learning	Diagnostic accuracy of the *artificial neural network* machine learning tool was 94.2%
Karaman et al. [30]	2025	Turkey	Retrospective cohort study	Adult participants *n* = 124 Males = 73/124 Females = 51/124	35.26 +/− 14.30	Retinitis pigmentosa	Multifocal ERG	Machine learning	Highest diagnostic accuracy by *support vector machine* machine learning at 87.1%
Karaman et al. [31]	2025	Turkey	Retrospective cohort study	Human participants *n* = 97 Males = 61/97 Females = 36/97	37.48 +/− 16.19	Retinitis pigmentosa	Multifocal ERG	Machine learning	Highest diagnostic accuracy was by *naïve Bayes* machine learning at 82.32%
Kulyabin et al. [32]	2023	Germany; Russia	Retrospective cohort study	Paediatric participants *n* = unstated	Unstated	Healthy and unhealthy, unstated specific diagnosis	Full-field ERG	Deep learning	Highest diagnostic accuracy was by *vision transformer with ricker wavelet* deep learning at 84.0% for maximum ERG response, 84.9% for scotopic ERG response, 87.5% for photopic ERG response
Kulyabin et al. [33]	2023	Russia	Prospective cohort study	Adult and paediatric participants *n* = 323	Unstated	Healthy and unhealthy, unstated specific diagnosis	Full-field ERG	Deep learning	Highest diagnostic accuracy was by *visual transformer small* deep learning at 88.0% for maximum ERG response, 85.0% for scotopic ERG response, 91% for photopic ERG response
Zhdanov et al. [34]	2022	Russia; Germany	Retrospective cohort study	Adult and paediatric participants *n* = 103 Adult = 38 Paediatric = 65	Unstated	Healthy and unhealthy, unstated specific diagnosis	Full-field ERG	Machine learning	Highest diagnostic accuracy was by *classical features + wavelet 1–4* machine learning at 83% in the adult group. High diagnostic accuracy was by “wavelet 1–2” machine learning at 70% in the paediatric group
Zhdanov et al. [35]	2023	Russia; Germany; Romania	Retrospective cohort study	Adult and paediatric participants *n* = unstated	Unstated	Healthy and unhealthy, unstated specific diagnosis	Full-field ERG	Machine learning	Diagnostic accuracy of the “decision trees” machine learning tool was 52% in the adult group and 40% in the paediatric group

## Data Availability

No new data were created or analysed in this study. Data sharing is not applicable to this article.

## Supplementary Materials

The following supporting information can be downloaded at: https://www.mdpi.com/article/10.3390/ijms27083491/s1. Reference [17] is cited in the Supplementary Materials.

## Appendix A. Complete Search Strategy Across Five Databases

PubMed:

((electroretinography[MeSH Terms]) OR (electroretinographies[MeSH Terms]) OR (electroretinography[Title/Abstract]) OR (electroretinographies[Title/Abstract]) OR (electroretinogram[Title/Abstract])).

AND

((ai artificial intelligence[MeSH Terms]) OR (artificial intelligence[Title/Abstract]) OR (AI[Title/Abstract]) OR (active machine learning[MeSH Terms]) OR (algorithm, machine learning[MeSH Terms]) OR (deep learning[MeSH Terms]) OR (computational neural network[MeSH Terms]) OR (computational neural networks[MeSH Terms]) OR (machine?learning[Title/Abstract]) OR (deep?learning[Title/Abstract])).

Medline:

(MH “Artificial Intelligence+”) OR (MH “Intelligent Systems”) OR (MH “Machine Learning+”) OR (MH “Deep Learning+”) OR (MH “Large Language Models”) OR (MH “Multifactor Dimensionality Reduction”) OR (MH “Ensemble Learning+”) OR (MH “Federated Learning”) OR (MH “Reinforcement Machine Learning”) OR (MH “Representation Machine Learning”) OR (MH “Supervised Machine Learning+”) OR (MH “Support Vector Machine”) OR (MH “Transfer Machine Learning”) OR (MH “Unsupervised Machine Learning+”) OR (MH “Particle Swarm Optimization”) OR (MH “Pattern Analysis, Machine”) OR (MH “Prediction Methods, Machine+”) OR (MH “Predictive Learning Models”) OR (MH “Sentiment Analysis”).

AND

(MH “Electroretinography”) OR “electroretinograph*” OR “electroretinogram*”.

Embase:

‘electroretinography’:ti,ab,kw OR ‘electroretinogram’:ti,ab,kw OR’electroretinographies’:ti,ab,kw).

AND

(‘artificial intelligence’:ti,ab,kw OR ‘machinelearning’:ti,ab,kw OR ‘deep learning’:ti,ab,kw) OR (‘artificial intelligence’/exp OR ‘artificial intelligence’ OR ‘artificial intelligence-assisted technology’/exp OR ‘artificial intelligence-assisted technology’) AND(‘electroretinography’ OR ‘electroretinogram’/exp OR ‘electroretinogram’ OR’electroretinograph’/exp OR ‘electroretinograph’).

Scopus:

TITLE-ABS-KEY (electroretinography OR electroretinographies OR electroretinogram) AND TITLE-ABS-KEY (“artificial intelligence” OR “AI” OR “machine learning” OR “deep learning”) AND (LIMIT-TO (DOCTYPE, “ar”)).

Web of Science:

electroretinography OR electroretinographies OR electroretinogram (Topic) and artificial intelligence OR AI OR machine learning OR deep learning (Topic).
